# Planning for operating room efficiency and faster anesthesia wake-up time in open major upper abdominal surgery

**DOI:** 10.1097/MD.0000000000006148

**Published:** 2017-02-17

**Authors:** Hou-Chuan Lai, Shun-Ming Chan, Chueng-He Lu, Chih-Shung Wong, Chen-Hwan Cherng, Zhi-Fu Wu

**Affiliations:** a Department of Anesthesiology, Tri-Service General Hospital and National Defense Medical Center; b Division of Anesthesiology, Cathay General Hospital, Taipei, Taiwan, Republic of China.

**Keywords:** anesthesia-controlled time, desflurane, open major upper abdominal surgery, propofol

## Abstract

Reducing anesthesia-controlled time (ACT) may improve operation room (OR) efficiency result from different anesthetic techniques. However, the information about the difference in ACT between desflurane (DES) anesthesia and propofol-based total intravenous anesthesia (TIVA) techniques for open major upper abdominal surgery under general anesthesia (GA) is not available in the literature.

This retrospective study uses our hospital database to analyze the ACT of open major upper abdominal surgery without liver resection after either desflurane/fentanyl-based anesthesia or TIVA via target-controlled infusion with fentanyl/propofol from January 2010 to December 2011. The various time intervals including waiting for anesthesia time, anesthesia time, surgical time, extubation time, exit from OR after extubation, total OR time, and postanesthetic care unit (PACU) stay time and percentage of prolonged extubation (≥15 minutes) were compared between these 2 anesthetic techniques.

We included data from 343 patients, with 159 patients receiving TIVA and 184 patients receiving DES. The only significant difference is extubation time, TIVA was faster than the DES group (8.5 ± 3.8 vs 9.4 ± 3.7 minutes; *P* = 0.04). The factors contributed to prolonged extubation were age, gender, body mass index, DES anesthesia, and anesthesia time.

In our hospital, propofol-based TIVA by target-controlled infusion provides faster emergence compared with DES anesthesia; however, it did not improve OR efficiency in open major abdominal surgery. Older, male gender, higher body mass index, DES anesthesia, and lengthy anesthesia time were factors that contribute to extubation time.

## 1 Introduction

Anesthesia-controlled time (ACT) and turnover time are 2 of the most important factors that regulate operation room (OR) efficiency.^[1]^ Extubation time is of special interests to surgeons and anesthesiologists because it could be affected by different anesthetic agents or techniques.^[2–4]^ Prolonged extubation is an important factor that would decrease OR efficiency. Prolonged extubation time would cause slowing of work flow, having OR members staying idly waiting for extubation, and the surgeon have to wait longer for next operation. Surgeons always want patient quick to awaken.^[5]^ Accordingly, choosing appropriate anesthetic agents or techniques to avoid prolonged extubation is essential for anesthesiologists in order to improve the efficiency of OR. Dexter and Epstein^[6]^ recommended that recording extubation time and monitoring the incidence of prolonged extubation is very important especially at facilities that have at least 8 hours of cases and turnovers.

The ACT between total intravenous anesthesia (TIVA) with propofol and desflurane (DES) anesthesia was investigated, nevertheless, the results are controversial.^[4,7–15]^ The majority of these studies comparing the effects of different anesthesia regimens on OR efficiency have tended to focus on ambulatory or short-time surgery. As our best knowledge, we found no comparisons in different anesthetic techniques for the improvement of ACT in open major upper abdominal surgery under general anesthesia (GA). Moreover, different propofol delivery techniques such as target-controlled infusion (TCI) and syringe pump infusion were used in these studies and may lead to different results. The aim of our present study was to determine whether the use of TIVA with TCI system is more effective than DES anesthesia in reducing ACT in patients undergoing open major upper abdominal surgery.

## 2 Methods

This retrospective study was approved by the Ethics Committee (TSGHIRB No: 100-05-168) of Tri-Service General Hospital, Taipei, Taiwan (Chairperson, Professor Pauling Chu) on August 29th, 2011. IRB allows waiving the requirement for obtaining informed consent, and patient records were anonymized and deidentified prior to analysis. The information was retrieved from the medical records and the electronic database of Tri-Service General Hospital (TSGH; Taipei, Taiwan, Republic of China). We enrolled 343 patients (American Society of Anesthesiology class I–III) who received elective open major upper abdominal surgery under TIVA with TCI or DES anesthesia from January 2010 to December 2011. Exclusion criteria include: body mass index (BMI) >35 kg/m^2^, liver resection, emergent surgeries, patient's age younger than 18 years, combined TIVA with inhalation anesthesia or epidural anesthesia, other inhalation anesthesia besides DES, patients were sent to the intensive care unit, or incomplete data. Other parameters included demographic data and American Society of Anesthesiology physical status.

There was no premedication before induction of anesthesia. Regular monitoring, such as noninvasive blood pressure, arterial line, electrocardiography (lead II), pulse oximetry, and end-tidal carbon dioxide (EtCO_2_) pressure, was applied in each patient. Anesthesia was induced with fentanyl, propofol, and rocuronium in all patients. The patients were then intubated and maintained with propofol or DES and the analgesic fentanyl. In our common practice, we take patients to the postanesthetic care unit (PACU) after extubation and did not extubate in PACU.

In the TIVA group, anesthesia was induced using intravenous (i.v.) fentanyl (2 μg/kg) and 2% lidocaine (1.5 mg/kg). Continuous infusion of propofol (fresfol 1%) was delivered subsequently using Schneider kinetic model of TCI (Fresenius Orchestra Primea; Fresenius Kabi AG, Bad Homburg, Germany) with the effect-site concentration (Ce) of 4.0 μg/mL. Rocuronium (0.6 mg/ kg) was administered when patients lost consciousness, followed by tracheal intubation. Anesthesia was maintained using TCI with propofol Ce 3 to 4 μg/mL and an oxygen flow of 0.3 L/min. Repetitive bolus injections of cisatracurium and fentanyl were prescribed as required throughout the procedure.^[12,16]^

In the DES group, the patients were induced with i.v. fentanyl (2 μg/kg), 2% lidocaine (1.5 mg/kg), and propofol (1.5–2 mg/kg). When patients lost consciousness, 0.6 mg/kg of rocuronium was administered, followed by endotracheal intubation. Anesthesia was maintained using 8% to 12% DES (inhaled concentration) in an oxygen flow of 300 mL/min under a closed system without nitrous oxide. Repetitive bolus injections of cisatracurium and fentanyl were prescribed as required throughout the procedure.^[12,16]^

Maintenance of the Ce for the TCI with propofol and DES concentration was adjusted at the range of 0.2 μg/mL and 0.5%, respectively, according to the hemodynamics. If 2 increments or decrements were unsuccessful, the range of Ce for TCI propofol and DES was increased to 0.5 μg/mL or 2%, respectively. The EtCO_2_ pressure was maintained at 35 to 45 mm Hg by adjusting the ventilation rate and maximum airway pressure. Once neuromuscular function returns, cisatracurium (2 mg, i.v.) was administered as required.^[12,16]^

Ce of propofol or DES concentration was tapered to 2.0 μg/mL or 5% respectively at the beginning of skin closure. At the last 5 stitches of surgery, propofol or DES was discontinued, but the oxygen flow was kept 300 mL/min. At the end of the skin closure, the lungs were ventilated with 100% oxygen at a fresh gas flow of 6 L/min. Reversal of neuromuscular function was achieved by administrating neostigmine (0.03–0.04 mg/kg) with glycopyrrolate (0.006–0.008 mg/kg) once spontaneous breathing returned to prevent residual paralysis. When the patient regained consciousness by name with spontaneous and smooth respiration, the endotracheal tube was removed and the patient was sent to the PACU for further care. An extubation time (from the end of skin closure until extubation) equal or longer than 15 minutes is considered prolonged extubation.^[17]^

Data are presented as the mean and standard deviation, number of patients, or percentage. Demographic and perioperative variables were compared using Student *t* tests. Categorical variables were compared using chi-square test. Multivariable logistic regression analyses were performed to assess the association between variables contributed to prolonged extubation. The level of statistical significance was determined as *P* < 0.05.

## 3 Results

**Figure 1:**
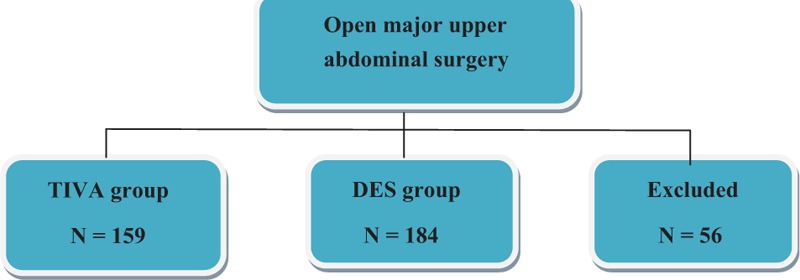
The flow diagram. DES = desflurane anesthesia, TIVA = total intravenous anesthesia.

After excluded from the electrical record, another 56 patients were excluded from the analysis. Of those excluded, 20 patients received combined inhalation anesthesia with propofol and 15 patients received sevoflurane anesthesia, and 21 patients had BMI >35 kg/m^2^ (Fig. 1).

Our study included 343 patients, of which 184 received DES and 159 received TIVA anesthesia. Summary of surgical procedures was shown in Table 1. There was no significant difference in patient demographics (Table 2). The amount of opioids and nondepolarizing muscle relaxants (NDMRs) were significant higher in TIVA group than in DES group while reversal agents showed no significant difference between groups (Table 3). The emergence was faster for TIVA group than DES group (8.5 ± 3.8 vs 9.4 ± 3.7 minutes; *P* = 0.04). The waiting for anesthesia time, surgical time, anesthesia time, exit from OR after extubation, total OR time, PACU time, and the incidence of prolonged extubation were no difference between groups (Table 4).

**Table 1:**
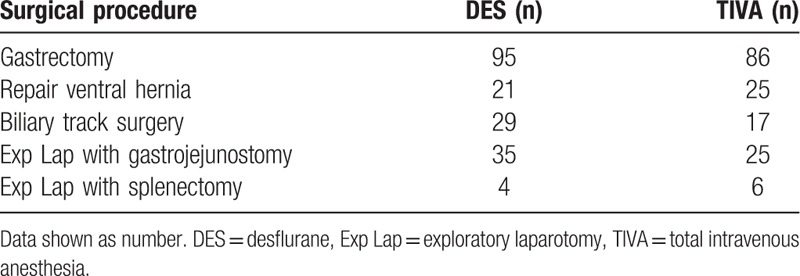
Summary of surgical procedures.

**Table 2:**
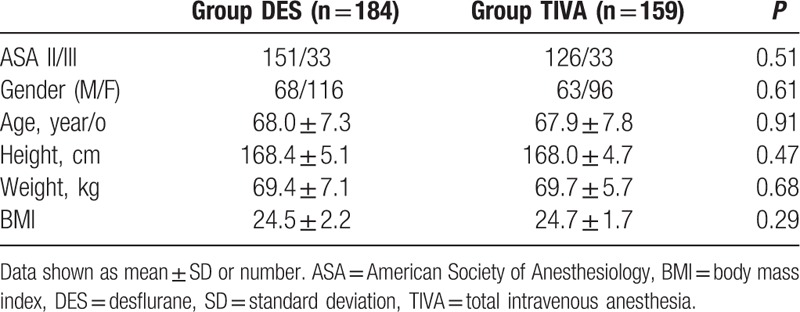
Patient's characteristics.

**Table 3:**
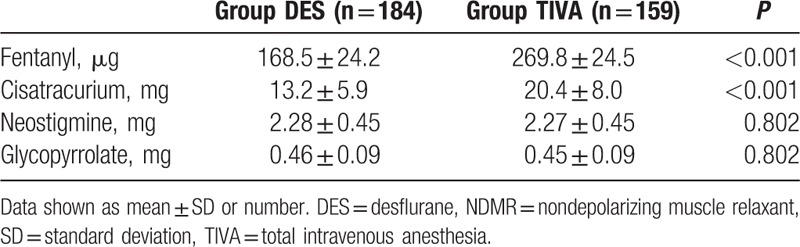
The amount of opioid, NDMRs, and reversal agents during surgical periods between DES and TIVA group.

**Table 4:**
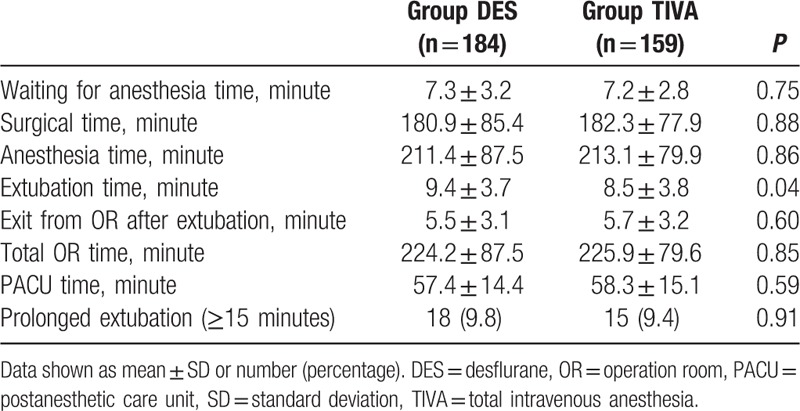
OR time measurement between DES and TIVA group.

The result of multivariable logistic regressions comparing prolonged extubation time between several variants in all patients is shown in Table 5. Age, gender, BMI, group, and anesthesia time were factors that contribute to extubation time. The results showed that patients with older age, male, higher BMI, DES anesthesia, and lengthy anesthesia time have slower emergence.

**Table 5:**
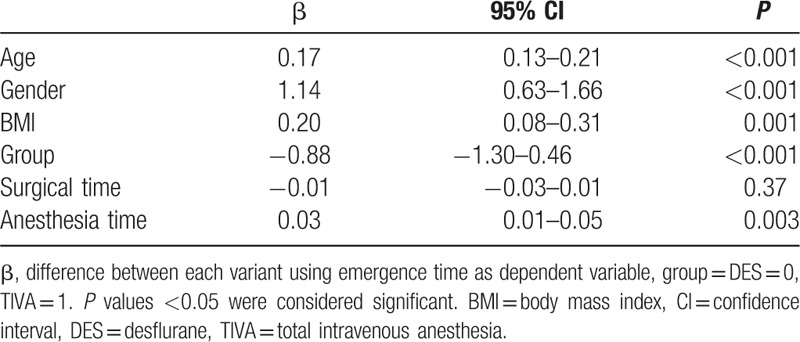
Multivariable linear regression analyses of variables associated with extubation time in all patients (n = 343).

## 4 Discussion

The major findings in this retrospective study show that propofol-based TIVA by TCI reduced the extubation time relative to DES anesthesia. Although statistically significant differences were found, 0.9 minutes reduction in extubation time in TIVA group suggested less clinical or economical effect on the ACT component of OR efficiency. In addition, we found that the factors of prolonged extubation are age, gender, BMI, DES group, and anesthesia time in patients undergoing open major upper abdominal surgery.

The 1st finding was consistent with several previous studies showing that GA using TCI system with propofol could achieve faster extubation than using DES in different surgeries.^[9–12,18]^ In our previous large case number retrospective studies, we showed that propofol-based TIVA by TCI reduced the extubation time were1.8 and 5.4 minutes relative to DES in patients undergoing ophthalmic surgery^[12]^ and lengthy lumbar spine surgery.^[13]^ Because the awakening time can be predicted by TCI system.^[19]^ However, 4 studies compared DES anesthesia with propofol-based TIVA and failed to show any significant clinical difference in extubation in laparoscopic cholecystectomy,^[20]^ otological surgery,^[21]^ ear, nose, and throat surgery.^[22]^ These were different from our retrospective studies and other previous studies.^[9,11,12,18]^ The reason might be due to the DES maintenance flow rate of oxygen was different: 1 to 4 L/min versus 300 mL/min in our study. Using close circuit anesthesia in the DES group would also prolong the neuromuscular blockade and delay the extubation time.^[23]^ In another study, Dolk et al^[24]^ had reported that there were shorter extubation time for DES anesthesia compared with propofol delivered by TCI in knee surgery. The difference may cause by using nitrous oxide as an adjuvant to anesthetics, which reduce the requirement of DES during the maintenance period and facilitate early emergence.

Epstein et al^[25]^ concluded that prolonged extubation time should result in increased variable costs. Another study conducted by the same group demonstrated that the mean time from end of surgery to exit OR is at least 12.6 minutes longer in cases with prolonged extubation and that the percentage of cases for which the extubation was prolonged among anesthesia for intraperitoneal procedures in upper abdomen was 15.1% ± 0.6%.^[6]^ In our present study, the percentage of prolonged extubation in DES group is 9.8%, while the percentage of prolonged extubation in TIVA group was 9.4% (Table 4). There was no significant difference in the incidence of prolonged extubation between TIVA and DES groups, which might be due to the similar BMI, gender, surgical time, and anesthesia time.

There were studies that investigated the confounding risk factors of prolonged extubation which included prone position, prolonged surgical time, significant blood loss, and larger volume of crystalloid and colloid infusion.^[6,26,27]^ Our previous study reported that DES anesthesia, lengthy anesthesia time, higher BMI, and shorter surgical time contribute to slower emergence in gynecologic laparoscopic surgery.^[14]^ In addition, Chan et al^[19]^ demonstrated that the confounding factors that predicted awaken under TCI with propofol are age, gender, and times of surgery and anesthesia (total consumption dose of propofol and fentanyl) in assortments of surgeries. In this study, old age, male gender, higher BMI, and lengthy anesthesia time resulting in prolonged extubation, which was consistent with our previous studies.^[14,19]^ Nevertheless, we showed that surgical time did not contribute to prolonged extubation, it might be due to the prolonged duration of neuromuscular relaxants resulting from the close circuit anesthesia.^[23]^

Previous studies also implied that longer-than-average anesthesia times strongly influence the academic anesthesiology departments by increasing the staffing costs and decreasing hourly productivity.^[28,29]^ There is evidence that propofol may accumulate and redistributed from the fatty tissue and muscle to the plasma, which leads to delay recovery by using syringe pump with continuous infusion in adult.^[30]^ However, TCI could maintain the steady concentration of propofol instead of flow rate and predicit awake time. Therefore, the effect of accumulation and redistribution of propofol on extubation should be less than syringe pump with continuous infusion of propofol. The inhaled DES is redistributed in the fatty tissue and muscle and delayed emergence once the lengthy anesthesia time.^[18]^ Therefore, monitoring anesthetic depth such as bispecrtal index (BIS) to keep the hypnotic level within the recommended range improves anesthetic delivery and postoperative recovery from relatively deep anesthesia.^[31]^ In addition, we suggested prescribed BIS for patients were elder, higher BMI, and lengthy anesthesia time.

The amount of opioid and NDMRs in DES group was significantly lower than in TIVA group during surgical periods. It is reasonable because volatile anesthetics may increase the potency of NDMRs^[32]^ and demonstrate synergy effects with opioids.^[33]^ In addition, not until spontaneous breathing returned were the reversal agents administrated. Therefore, we believed the final neuromuscular blockade status and amount of reversal agents given were matched between groups.

Our previous studies showing that GA using TCI system with propofol could achieve faster extubation than using DES anesthesia in different surgeries.^[9–14,18]^ Different anesthetic manipulations before emergence in various types of surgical procedures might explain the differences in findings. For example, in breast^[11]^ and gynecologic surgery,^[14]^ propofol was adjusted to a Ce of 2.0 μg/mL and the vapor of DES was changed to 5.0% in the beginning of wound closure. After gauze coverage, propofol and DES were discontinued and lungs were ventilated with 100% oxygen at a gas flow of 6 L/min. In ophthalmic surgery,^[12]^ DES or propofol was discontinued after the surgery, and the lungs were ventilated with 100% oxygen at a fresh gas flow of 6 L/min. In spine surgery, we discontinued DES or propofol at the end of the operation or at the last 3 stitches of surgery. After turning the patients to supine position, the lungs were ventilated with 100% oxygen at a fresh gas flow of 6 L/min.^[10,13]^ In addition, we used closed-circuit anesthesia in the DES patients, which would prolong neuromuscular blockade and contribute to delay emergence.^[23]^

There are many limitations in the study. The first was our study is a retrospective study. Considering comparability and standardization of study groups, a retrospective study may contribute to bias. Although the choice of anesthetic management was not randomly allocated but rather by the availability of the TCI devices, the results showed no difference in the characteristics of the patients between 2 groups. The study, performed under clinical conditions and provided large sample size, reflects more precisely the clinical relevant benefit. Second, we excluded liver resection due to liver dysfunction resulting in major impacts on the pharmacokinetics and pharmacodynamics of anesthetics and the recovery from TIVA and inhalation anesthesia is delayed in hepatectomy patients.^[34,35]^ Third, we did not compare the effect of body temperature on extubation time, because hypothermia may delay awakening.^[36]^ However, in our cases, we used the patient warming system including fluid warming kit and convective air warming system to keep their core temperature ≧35 °C perioperatively. Fourth, we excluded patients with BMI >35 kg/m^2^, because obesity may lead to prolonged extubation^[36]^ and it is the limitation in Schnider model of TCI machine. Fifth, we did not include patients receiving Whipple operation and blood loss >1500 mL because larger volume of fluid infusion may be the risk factor for delayed extubation.^[26]^ Finally, we did not use BIS in our common practice. But the depth of anesthesia was monitored by the experienced anesthesiologist, and our percentage of prolonged extubation was 9.6% less than overall 15.4% reported by a previous study.^[6]^

Although anesthesia has the capacity to reduce operating room efficiency, a well-planned anesthesia technique like propofol-based TIVA or DES does not impede efficiency even after lengthy invasive surgery and even in an academic teaching hospital setting. Therefore, other factors (adequate preoperative patient workup, hospital transport, preparation in the preanesthesia unit, surgical time, patient comorbidities, etc.) need to be considered.
